# Immunomodulatory potential of polysaccharides from *Coriolus versicolor* against intracellular bacteria *Neisseria gonorrhoeae*

**DOI:** 10.14202/vetworld.2019.735-739

**Published:** 2019-06-01

**Authors:** Manikya Pramudya, Sri Puji Astuti Wahyuningsih

**Affiliations:** Department of Biology, Faculty of Science and Technology, Universitas Airlangga, Surabaya, Indonesia

**Keywords:** immune response, immunomodulator, *Neisseria gonorrhoeae*, polysaccharide

## Abstract

**Background and Aim:**

For many years, people use natural products from the plant and fungal to improve immune response against microorganism. This study aimed to investigate the immunomodulatory properties of polysaccharides (PS) from *Coriolus versicolor* in mice infected by intracellular bacteria *Neisseria gonorrhoeae*.

**Materials and Methods:**

Thirty-six female BALB/C mice were divided into six groups: Normal control, negative control, positive control, P1 (PS before infection), P2 (PS after infection), and P3 (PS before and after infection). PS were administrated for 10 days. *N. gonorrhoeae* was infected twice with 2 weeks gap from the first to second exposure with a dose of 10^6^ cells. 1 week after the end of treatment, level of oxidants, innate immune responses, and adaptive immune responses were measured.

**Results:**

This study showed that PS administration could restore the number of leukocytes as normal but could not enhance the number of phagocytes and its activity. PS administration also showed immunosuppression activity by lowering nitric oxide levels in P2 and P3 groups (p<0.05). This result showed that PS prevent over-expression of pro-inflammatory cytokines by decreasing phagocytic activity. Contrast with innate immune response result; PS administration could significantly increase interferon-gamma level in P1, P2, and P3 groups (p<0.05). Level of antibodies was significantly increased in the P3 group (p<0.05). PS administration also showed an increased level of tumor necrosis factor-α, but the difference was not significant (p>0.05).

**Conclusion:**

PS enhance adaptive immunity due to the capability of *N. gonorrhoeae* that able to survive and replicate in phagocytes. Thus, PS from *C. versicolor* could be potentially be used as a natural immunomodulator against intracellular bacteria.

## Introduction

Sexually transmitted infection such as gonorrhea is one of the major public health problems which has been causing serious morbidity and mortality [1]. *Neisseria gonorrhoeae* infections are approximately 60 million cases each year worldwide [2]. Hananta *et al*. [3] reported that the prevalence of asymptomatic urogenital gonorrhea among the Indonesian population is very high. *N. gonorrhoeae* infects the female cervix and male urethra leads to severe complications such as pelvic inflammatory disease, urethritis, cervicitis, ectopic pregnancy, disseminated gonococcal infection, and infertility [4]. Moreover, *N. gonorrhoeae* infection is associated with increased risk of HIV transmission [5]. *N. gonorrhoeae* are intracellular bacteria that able to survive and replicate inside of the cell [6]. Naturally, innate immunity can inhibit transmission of *N. gonorrhoeae*, but these bacteria are relatively resistant to degradation of phagocytes and able to modulate apoptosis in macrophage [7,8]. Immune response mediated by T-cell is needed to destroy these bacteria. The human body needs a particular compound to modulate immune response.

Polysaccharides (PS) from natural sources such as fungal and plant have been known to improve the immune function of the body [9,10]. *Coriolus versicolor* is one of the medicinal mushrooms used in Japan, China, Korea, and other Asian countries [11]. Both PS krestin and PS peptide from *C. versicolor* are active as biological response modifier. Carbohydrate in powdered polysaccharide krestin contains 91-93% active compound β-glucan [11]. Wahyuningsih *et al*. [12] reported that PS of *C. coriolus* could enhance the response of immunoglobulin against *Pseudomonas aeruginosa*. Another study was also reported that polysaccharide from *C. versicolor* induce B-cell activation and enhance cytokine production [13]. However, studies have not been reported on the activity of PS from *C. coriolus* as an immunomodulator against *N. gonorrhoeae* in Indonesia.

Therefore, this study aimed to investigate the immunomodulatory properties of PS from *C. versicolor* growth in Indonesia including number of phagocytes, number of leukocytes, phagocytic activity, level of cytokine, level of antibody, and level of nitric oxide (NO) in mice infected by intracellular bacteria *N. gonorrhoeae*.

## Materials and Methods

### Ethical approval

All procedures involving animal care were carried out in accordance with the guidelines laid down by Animal Care and Use Committee of Veterinary Faculty, Universitas Airlangga, Surabaya, Indonesia.

### Materials and chemicals

*C. versicolor* was collected from Surabaya, Kediri and Tulungagung, East Java, Indonesia. *N. gonorrhoeae* was purchased from Balai Besar Laboratorium Kesehatan, Surabaya Indonesia. Antibody level, interferon-γ (IFN-γ), tumor necrosis factor (TNF)-α, and enzyme-linked immunosorbent assay (ELISA) kit were purchased from BioLegend (BioLegend, Inc., San Diego, USA). All other chemicals and solvent used were of analytical reagent grade.

### Preparation of crude PS from *C. versicolor*

According to Cui and Christi [11], *C. versicolor* in coarse powder (200 g) was macerated twice with 3 L and 1 L of water, heated at 80-98°C for 2-3 h. The sample was filtered using Whatman paper No.41, vacuum and Buchner funnel and supernatant were collected. The supernatants were precipitated by ammonium sulfate 90% for 24 h at 4°C. Then, the sample was centrifuged at 9000 rpm for 30 min at 4°C. The precipitated material was then dissolved in phosphate-buffered saline (PBS) (30 mL) and dialyzed through nitrocellulose membrane for 24 h in PBS at 4°C. The aqueous solution was freeze-dried to obtain polysaccharide from *C. versicolor* (PS).

### Animals

Thirty-six female BALB/c mice (8-10 weeks; 30-40 g) were obtained from Faculty of Pharmacy, Universitas Airlangga (Surabaya, Indonesia). The animals were maintained in cages made of plastic at 20°C, with 12-h light/12-h dark cycle, fed and watered by *ad libitum*.

### Experimental design

After 7 days of acclimatization, mice were randomly divided into six groups (KN: Normal control; K+: Positive control, K–: Negative control; P1: PS administration before infection; P2: PS administration after infection; and P3: PS administration before and after administration). PS (50 mg/kg BW) was administrated at 1-10 days and 26-36 days by gavage. Mice were exposed to *N. gonorrhoeae* (0.5 Mcfarland) twice at 11^th^ day and 25^th^ day. 1 week after the last administration, the mice were injected intraperitoneally with 0.2 mL of *Staphylococcus aureus* suspension. 1 h later, the mice were killed by ketamine anesthesia and 3 mL of 3% ethylenediaminetetraacetic acid was used as an anticoagulant. The intraperitoneal fluid was collected. Blood samples were also collected to obtain serum.

### Phagocytes and leukocytes counts

Blood sample (10 µL) and intraperitoneal fluid (30 µL) were dissolved with 100 µL of Turk solution, separately. Then, number phagocytes and leukocytes from both blood and intraperitoneal fluid were counted using hemocytometer.

### Phagocytes activity assay

The intraperitoneal suspension (70 µL) was smeared on glass slides and air-dried. The smear was fixed using methanol for 15 min and stained with Giemsa solution for 20 min. Phagocytic activity was determined by counting the number of phagocytes in a population of 100 phagocytes.

### Serum cytokines and antibody assay

Whole blood was collected and centrifuged at 3000 rpm and 4°C for 10 min, while the upper layer contained the serum. The levels of antibody, IFN-γ, and TNF-α, in the serum, were analyzed by commercial ELISA kits (BioLegend, Massachusetts, USA) according to the manufacturer’s protocol. Level of cytokines IFN-γ and TNF-α was measured using Sandwich-ELISA method. Meanwhile, the level of antibody was measured using an indirect ELISA method. The absorbance was measured using the ELISA reader at 450 nm.

### NO assay

Deproteinated serum (50 µL) was added with 100 µL Griess reagent I and 100 µL Griess Reagent II. After that, the sample was homogenized using vortex and incubated for 10 min at room temperature. The absorbance was measured at 540 nm.

### Statistical analysis

Statistical analysis was performed by one-way analysis of variance followed by Duncan’s *post hoc* test. All analyses were performed using SPSS Statistic 24 Software (IBM Corporation, USA). The results were reported as the mean±standard deviation of six repeats. p<0.05 was considered statistically significant.

## Results

### Number of phagocytes

**Figure-1:**
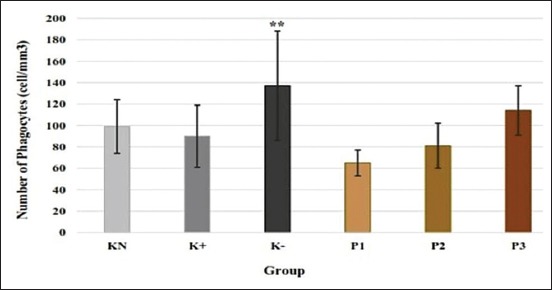
Effect of polysaccharides from *Caribena versicolor* on a number of phagocytes (cell×10^5^/mm^3^). KN=Normal control, K+=Positive control, K−=Negative control, P1=Administration of polysaccharides (PS) before infection, P2=Administration of PS after infection, P3=Administration of PS before and after infection. Values are represented as means±standard deviation (n=6). **p<0.05 compared to KN group.

The number of phagocytes was significantly increased in KN–group compared to normal control (p<0.05). All of the treatment groups with the administration of *C. versicolor* PS showed no significant difference compared to control groups. P3 group (114±23 cell/mm^3^) showed an increase of phagocytes, but the difference was not significant (Figure-1).

### Number of leukocytes

**Figure-2:**
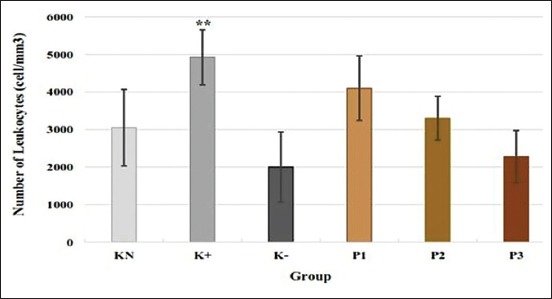
Effect of polysaccharides from *Caribena versicolor* on a number of leukocytes (cell/mm^3^). KN=Normal control, K+=Positive control, K−=Negative control, P1=Administration of polysaccharides (PS) before infection, P2=Administration of PS after infection, P3=Administration of PS before and after infection. Values are represented as means±standard deviation (n=6). **p<0.05 compared to KN group.

The highest number of leukocytes was shown by K+ group (4430±735 [cell/mm^3^]). K+ group also showed a significant increase in the number of leukocytes compared to normal control (p<0.05). P1 group (3560±860 [cell/mm^3^]) showed a higher number of leukocytes compared to normal control, but the difference was not significant (Figure-2).

### Phagocytic activity

**Figure-3:**
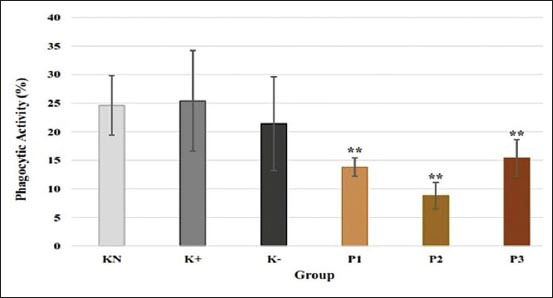
Effect of polysaccharides from *Caribena versicolor* on phagocytic activity (%). KN=Normal control, K+=Positive control, K−=Negative control, P1=Administration of polysaccharides (PS) before infection, P2=Administration of PS after infection, P3=Administration of PS before and after infection. Values are represented as means±standard deviation (n=6). **p<0.05 compared to KN group.

The highest phagocytic activity was shown in K+ group (25.4±8.8%). P1, P2, and P3 groups showed a significant decrease in phagocytic activity compared to the normal control group (p<0.05). Phagocytic activity of P1, P2, and P3 groups was 13.8±1.6%, 8.8±2.3%, and 15.4±3.2%, respectively (Figure-3).

### Level of antibody

P3 group showed a significant level of antibody compared to the normal control group (p=0.05). P3 group also had the highest level of antibody (0.498±0.048). P1 group showed an increase in the level of antibody, but the difference was not significant (Table-1).

**Table-1:** Effect of polysaccharide from *Caribena versicolor* on the level of antibody, IFN-γ, and TNF-α.

Group	Level of Antibody	Cytokine (pg/mL)	

		IFN-γ	TNF-α
KN	0.418±±0.051	39.5±22.8	842.7±245
K+	0.429±0.029	31.0±9.90	613.7±240.2
K−	0.484±0.041	136±103.9**	1276.4±453.9
P1	0.431±0.062	99.6±62.0**	1096.8±298.5
P2	0.403±0.071	206.7±146.6**	1239.6±363.6
P3	0.498±0.048**	735.0±130.7***	1036.2±359

KN=Normal control, K+=Positive control, K−=Negative control, P1=Administration of PS before infection, P2=Administration of PS after infection, P3=Administration of PS before and after infection, values are represented as means±SD (n=6).

** p<0.05 compared to KN group,

*** p<0.05 compared to all groups, TNF=Tumor necrosis factor, IFN=Interferon

### Cytokines production

Serum levels of IFN-γ were significantly increased in P1 group (99.6±62 pg/mL) and P2 group (206.7±146 pg/mL) compared to normal control and positive group (p=0.05). P3 group showed the highest level of IFN-γ (735±130.7 pg/mL) and was significantly different compared to all groups. Meanwhile, there was no difference in the result of the level of TNF-α. P1, P2, and P3 groups showed an increase in the level of TNF-α, but the difference was not significant (Table-1).

### NO

**Figure-4:**
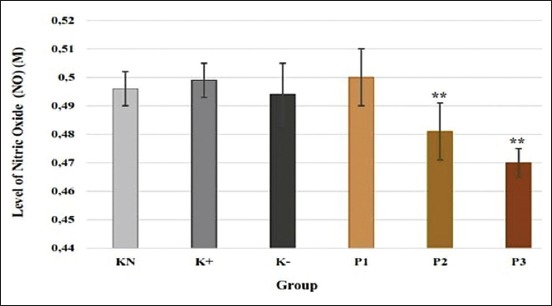
Effect of polysaccharides from *Caribena versicolor* on level of nitric oxide (M). KN=Normal control, K+=Positive control, K−=Negative control, P1=Administration of polysaccharides (PS) before infection, P2=Administration of PS after infection, P3=Administration of PS before and after infection. Values are represented as means±standard deviation (n=6). **p<0.05 compared to KN group.

P3 and P4 groups showed a significant decrease in the level of NO compared to the normal control group. NO level of P3 and P4 was 0.481±0.01 M and 0.470±0.005 M, respectively. NO level of P1 group was the same with normal control and did not show a significant difference (Figure-4).

## Discussion

Defense against microbes is mediated by innate immunity as the first response and the later response of adaptive immunity. The immune system offers a layered defense against microbes [7]. An unbalanced immune system against microbe will cause disease. Use of immunomodulator to enhance the host defense responses can be an effective way to increase resistance to disease [14]. PS have been regarded as important immunostimulant candidates. Besides plants PS, fungal PS are one of the promising natural immunomodulator and therapeutic agents [15,16].

Different microbes require a different mechanism for elimination. Adaptive immunity is able to differ bacteria compound from other microbes specifically. Immune system gives different response against intracellular and extracellular bacteria. *N. gonorrhoeae* is intracellular bacteria. Like most of the intracellular bacteria, *N. gonorrhoeae* enter the host through the mucosa [17]. The characteristic of these bacteria is able to survive and replicates within phagocytes. In this study, PS administration before and after infection, *N. gonorrhoeae* could not enhance number of phagocytes and number of leukocytes significantly. In line with that result, this study also showed a decrease in phagocytic activity in all groups with the administration of PS.

Contrast with our result, Meng *et al*. [18] stated that polysaccharide krestin extracted from *C. versicolor* possessed to stimulate macrophage and phagocytes activity. *N. gonorrhoeae* as an antigen may able to resist phagocytic killing, escape the phagolysosome, and survive inside macrophages [8]. Our results demonstrated that polysaccharide from *C. coriolus* might increase adaptive immune cells responses.

Murphy and Weaver [19] stated that the accumulation of *N. gonorrhoeae* activates sensor cells of the innate immune system to trigger an adaptive immune response. Phagocytes as antigen presenting cell stimulate differentiation of Th-cell. Th-cell that specifically gives response to the infection of intracellular bacteria is Th-1 cells [20]. Related with immunity, cytokines are important compound that mediates many cellular reactions. Cytokines are small protein produced by immune cells such as macrophages, T-helper cell, and NK cells [21]. In this study, we examine the level of TNF-α and IFN-γ. The present result showed that level of IFN-γ in all groups with the administration of PS before, after, and before-after infection was increased significantly. Other studies also reported that PS from *I. obliquus* and *C. versicolor* significantly increased secretion of IFN-γ [22,18]. Meanwhile, TNF-α level was increased but did not show a significant difference. This result showed that PS from *C. versicolor* could enhance cytokines production.

According to Abbas *et al*. [7], *N. gonorrhoeae* as intracellular bacteria is resistant to phagocytosis. Adaptive immune compounds are needed to eliminate it. The statement is in line with this study. This study showed a decrease in phagocytic activity in all treatment groups. Meanwhile, there was increase in production of TNF-α and IFN-γ. Our result showed that *C. versicolor* modulates immune system through the production of cytokines, which lead to activation of adaptive immune cells.

TNF-α and IFN-γ are cytokines produced by macrophage and Th cell due to all kinds of antigen infections. In this study, TNF-α and IFN-γ were not dominantly produced by macrophages because there is no relationship between phagocytic activity with the production of TNF-α and IFN-γ. Based on D’Elios [20], Th-1 cell also produced a high level of TNF-α and IFN-γ. Wahyuningsih *et al*. [23] also reported that TNF-α was not produced by macrophages during phagocytosis in the study using okra PS.

We found that the insignificant result of the increase in TNF-α level was beneficial. TNF-α is one of the important pro-inflammatory cytokines. The overproduction of TNF-α will induce the development of various disease and inflammation [24]. Insignificant result of TNF-α in line with decreases level of NO. NO is one of the important oxidative stresses released by macrophages. NO at a higher level will kill the normal cell and damage the tissue. PS also modulate the immune system by suppressing component of immune that leads to tissue damage.

Elimination of *N. gonorrhoeae* requires mechanism of cell-mediated immunity and adaptive immunity [7]. Activated Th-1 cell induces differentiation of B-cell to become plasma cell. Plasma cell produces antibody. Antibody enhances the lysis of these bacteria. This study showed that administration of PS from *C. versicolor* increases level of antibody significantly.

Immunomodulatory activities of PS from *C. versicolor* are due to the β-glucan compound. Chan *et al*. [25] stated that β-glucan recognizes and gives a response to bacterial infection faster. Active compound β-glucan is related with main immune cell receptor such as dectin-1 toll-like receptor-2/6 and complement receptor 3. β-glucan also able to stimulate the immune response of T-cell. According to Wang *et al*. [26], β-glucan in PS bound to dendritic cell-associated Dectin-1, leading to activation of protein tyrosine kinase, NF-κb signaling, and production of TNF-α.

Based on all of the result in this study, PS from *C. versicolor* could become the new candidate of immunomodulator. PS from *C. versicolor* enhance mainly adaptive immunity by increasing the production of cytokines and antibody. PS from *C. versicolor* also possessed immunosuppression activity by decreasing the level of phagocytic activity and NO to prevent tissue damage. Immunomodulation consist enhancement of the activity of immune cells, suppress component of immune that leads to tissue damage and restores the response of immune cells. In this study, PS from *C. versicolor* could do all immunomodulation mechanisms.

## Conclusion

We concluded that PS from *C. versicolor* could restore a number of leukocytes, increase antibody level, TNF-α level, and IFN-γ level but decrease phagocytic activity and NO level to prevent over-expression of pro-inflammatory cytokine. This study suggests that PS from *C. versicolor* could act as an effective compound to modulate the immune response.

## Authors’ Contribution

SPAW designed the study. MP performed the *in vivo* experiment and collected the samples. SPAW and MP analyzed the data. All authors drafted, read, and approved the final manuscript.
